# A Cohort Comparison of Lifespan After Age 100 in Denmark and Sweden: Are Only the Oldest Getting Older?

**DOI:** 10.1007/s13524-018-0755-7

**Published:** 2019-01-18

**Authors:** Anthony Medford, Kaare Christensen, Axel Skytthe, James W. Vaupel

**Affiliations:** 1 Interdisciplinary Center on Population Dynamics, University of Southern Denmark, Odense, Denmark; 2 Danish Aging Research Center, Department of Public Health, University of Southern Denmark, Odense, Denmark

**Keywords:** Centenarians, Increasing lifespan, Lifespan trends, Quantile regression

## Abstract

Although Denmark and Sweden have close cultural and historical ties, lifespans for Danes have generally been lower than those of Swedes. Recent improvements in Danish mortality after a period of stagnation have led to the suspicion that there may be positive trends at the very high ages at death within that population and that these trends could be quite different from those observed in Sweden. Although the mean ages at death for Danish and Swedish centenarians have been relatively constant at about 102 years for the cohorts born 1870–1904, the oldest-old in Denmark have been getting older, but no evidence has suggested any increase in lifespan for Swedes. Using quantile regression, we show that Danish centenarian lifespans in the 90th percentile have been lengthening, with those in 94th percentile (6 % longest-lived individuals) having a trend that is statistically significant at the 5 % level. We demonstrate that the increase observed is not due to the increasing sizes of birth cohorts and thus must be due to improving survival among this select top tier. We postulate that this super-select group in Denmark is best able to take advantage of the factors driving mortality reduction, whereas the majority of centenarians are not.

**Electronic supplementary material:**

The online version of this article (10.1007/s13524-018-0755-7) contains supplementary material, which is available to authorized users.

## Introduction

Globally, human longevity has been on the rise. Empirically, this has been observed from increases in life expectancy, increases in ages at death, and declines in death rates at older ages. At the population level, life expectancy at birth has been steadily increasing and has more than doubled in the last two centuries (Riley 2001). The rate of increase in its annual record has occurred at a pace of approximately three months per year for females for more than 150 years (Oeppen and Vaupel 2002) and indicates no signs of slowdown (White 2002). Whereas this increase in life expectancy was driven by reductions in infant mortality in earlier times, the main driver since about the 1950s has been reductions in mortality at older ages (Rau et al. 2008; Vaupel 1997; Vaupel et al. 1998).

One direct consequence of this is the rapid increase in—and accumulation of—the number of nonagenarians, centenarians, and supercentenarians (Maier et al. 2010; Vaupel and Jeune 1995). This phenomenon is particularly evident in the low-mortality countries of Europe (Robine and Paccaud 2005; Thatcher 2001), Japan (Robine and Saito 2003; Robine et al. 2003), and North America (Desjardins and Bourbeau 2010; Kestenbaum and Ferguson 2010), where the number of centenarians has been roughly doubling every decade since the 1960s (Vaupel and Jeune 1995).

At the individual level, the maximum observed individual lifespan (the time between birth and death) has been increasing steadily for more than one and a half centuries in Sweden, where high-quality data permit such observation (Wilmoth and Robine 2003; Wilmoth et al. 2000). This pattern is not limited to Sweden. In France, Japan, and England and Wales (and to a lesser degree, the United States), where data and record keeping are of a high quality, national demographic statistics also suggest a secular rise in the maximum age at death (Wilmoth and Lundström 1996).

In the literature, a common approach to the study of individual-level longevity improvement has been the analysis of time trends in the maximum observed ages at death using standard regression techniques. For example, Drefahl et al. (2012) did this for Sweden and remarked that, “Despite the stable [average] mortality pattern amongst Swedish centenarians, we saw a continued increase in the maximum age at death . . . .” This approach is problematic for two reasons. First, when regression methods are used, they are of the standard ordinary least squares (OLS) variety, which focuses on the mean of the lifespan distribution, conditional on some measure of time. However, the mean is a measure of central tendency, so the focus must be toward the center of the distribution. Because the real interest is in the right tail of the centenarian lifespan distribution, it would be more judicious and straightforward to examine the trends in that tail directly and not at the center of the distribution. Analyzing trends in the mean could be very misleading because trends over time for data at the center and at the tails of a distribution could be very different. Second, sole reliance on trends in maximum attained lifespan as indicators of longevity is fraught with pitfalls because increasing lifespan can be a simple artifact of increasing cohort sizes and not any underlying improvement in longevity. The maximum can increase over time solely because the centenarian population is growing. In order to circumvent these two issues—especially the theoretical issues around performing inference on the maxima—we propose a methodology not widely used in lifespan research.

Human mortality improvement can be studied in several distinct ways, and many summary mortality/longevity indicators are available; see, for example, Cheung et al. (2005) and references therein. Of these indicators, a comparatively small subset (Smith 1994) focused on the tails, and fewer still applied quantile-based measures. Examples of these include Vaupel (2003), who put forward an indicator called the *tail of longevity*, defined as the maximum attained age minus the age attained by the top 10th percentile of survivors. He also proposed the *relative tail of longevity*, which divides the tail of longevity by the top 10th percentile of survivors. Faber (1982) used the notion of *life endurancy*, defined as the age at which a specified proportion of births is still alive (e.g., the age reached by 1 in every 1,000 individuals). Life endurancy was found to increase substantially for both males and females in the United States between 1900 and 1990 and was projected to increase sharply by 2080 (Bell et al. 1992).

Quantile regression extends the simple quantile-based measures of survival to a more sophisticated regression context in which covariates can be included. The static quantile measures are made dynamic by using time as a covariate in the regression. The aim of this article is to demonstrate how quantile regression may be used to study lifespan trends. Several interesting questions can be considered. Has there has been any increase in centenarian lifespan over time? In other words, is there a positive and statistically significant relationship between lifespan and birth year? If a relation does exist, what is the strength of this relationship? Is this relationship the same at all parts of the lifespan distribution, or does it change with age? What is the strength of the relation at different points of the lifespan distribution? We limit our attention to trends in ages at death within a regression framework and therefore do not directly engage the ongoing debate about whether limits to lifespan exist. We use data from cohorts of Danish and Swedish centenarians and compare the two countries. Through a contrast of the results from Denmark and Sweden, some of the strengths of quantile regression should become clear.

Using quantile regression has advantages. Because it permits study of the entire lifespan distribution, one can assess trends across multiple quantiles simultaneously to gain a more complete picture of how the entire lifespan changes with time without restricting focus to the mean lifespan. Thus, trends in the average centenarian lifespan and in extreme lifespan (the tails) can be analyzed coherently within one framework. Given that our interest is in the tails, we can assess these directly by studying the high quantiles. By using high quantiles of the observed lifespan distribution, we avoid having to study the maximum ages at death, thereby sidestepping the use of more complicated inferential tools, such as extreme value theory. As far as we know, quantile regression has not previously been applied to the study of human lifespan trends.

## Data and Methods

For this type of analysis, high-quality individual-level data is paramount. This study is based on individual-level data from extinct cohorts in Sweden and Denmark. The data is obtained from the Danish Civil Register System (*Centrale Person Register* (CPR)) and the Swedish Total Population Register (*Registret över Totalbefolkningen* (RTB)). The Danish CPR was established in 1968, and all persons living in Denmark are registered for administrative use. It includes the name, gender, date of birth, place of birth, citizenship, unique personal identification number, and identity of parents; it also includes continuously updated information on vital status (i.e., whether dead, emigrated, disappeared, and so on), place of residence, and spouses (Pedersen 2011). The Church of Sweden has kept local registers of their parish members since the seventeenth century, but following national coverage and computerization, the RTB was established in its modern form in 1968. The Swedish register captures broadly similar information to the Danish one.

All persons in the analysis attained at least age 100 at death and were born between January 1, 1870 and December 31, 1904, amounting to 16,931 centenarians. Of these, 10,955 (8,772 females and 2,183 males) are Swedish, and 5,976 (4,681 females and 1,295 males) are Danish. Some basic descriptive statistics for the ages at death (all cohorts combined) are presented in Table 1.

**Table 1:** Basic descriptive statistics for the observed Danish and Swedish centenarian ages at death, cohorts 1870 to 1904 combined

	Denmark		Sweden	
	Female	Male	Female	Male
Number of Observations	4,681	1,295	8,772	2,183
Mean	101.93	101.68	101.94	101.68
Standard Deviation	1.74	1.47	1.77	1.57
Maximum	111.31	108.83	113.00	111.35

Koenker and Bassett (1978) developed the general theory of quantile regression, which extends the general regression framework to conditional quantiles of the response variable, such as the 99th percentile. Quantile regression is particularly useful when the rate of change in the conditional quantile, expressed by the regression coefficients, depends on the quantile. For a random variable *Y* with probability distribution function

$$ F(y)=P\left(Y\le y\right), $$

the τth quantile of *Y* is defined as the inverse function,

$$ {Q}_{\uptau}(Y)=\operatorname{inf}\left\{y:F(y)\ge \uptau \right\}, $$

where 0 < τ < 1 is the quantile level. For example, *Q*_0.5_ is the median; *Q*_0.75_ is the third quartile, or 75th percentile.

In our particular case, we fit, for any choice of quantile τ, the following model:

$$ {Q}_{\uptau}\left( Age\  at\  Death| Birth\ Year\right)={\upbeta}_0\left(\uptau \right)+{\upbeta}_1\left(\uptau \right)\times Birth\ Year. $$

The model coefficients are estimated using the *quantreg* package of the R statistical software program (R Core Team 2016). The online appendix (section A) presents details of the basic theoretical framework.

## Results

Because the male centenarian population is small both in absolute terms and relative to females, and the overall numbers dying at the highest ages are sparse, the analysis is performed with males and females combined. Before presenting the quantile regression results, we present the trends in the mean and the maximum ages at death both for motivation and comparison purposes.

**Fig. 1:**
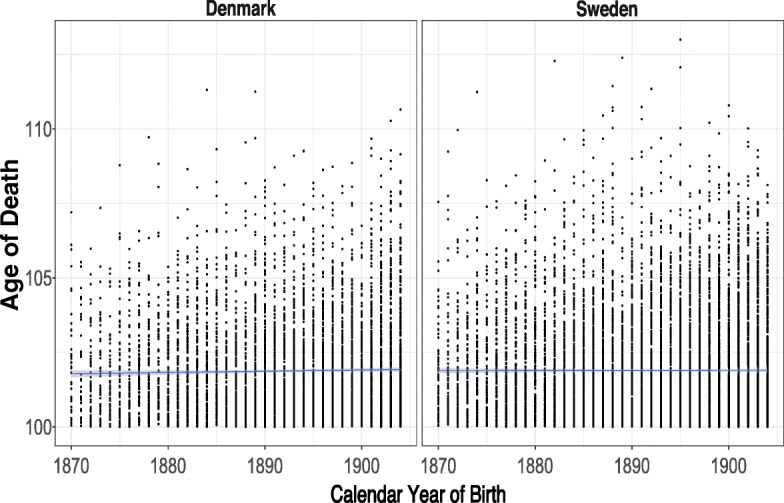
Age at death for Danish and Swedish female centenarian cohorts (1870–1904) with fitted linear trend and 95 % pointwise confidence bands. Each dot represents one individual.

Figure 1 indicates the ages of death by birth year together with a fitted OLS regression line. Visual inspection of the fitted line suggests that there is no secular upward trend in the ages at death of the centenarian cohorts.

**Fig. 2:**
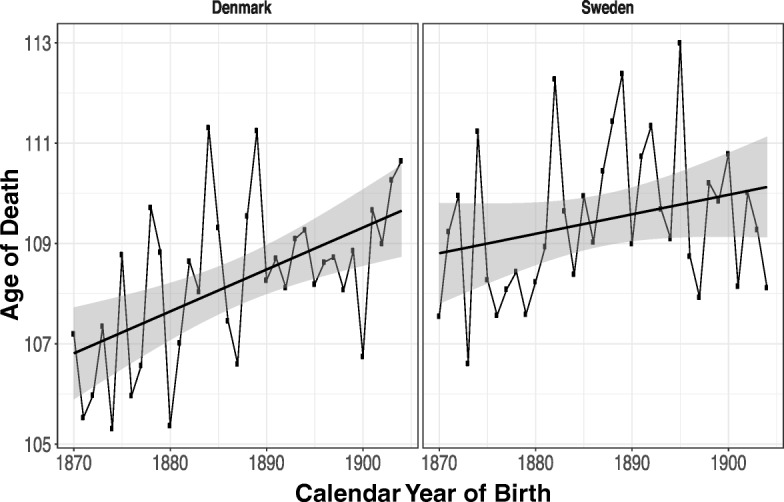
Maximum age at death for Danish and Swedish birth cohorts (1870–1904) with fitted linear trend and 95 % pointwise confidence bands. Each dot represents one individual.

The fitted means in both countries are roughly equivalent at approximately 102 years. For a more robust assessment of the presence of a trend in the ages at death, we conduct the Mann-Kendall test (Mann 1945). The *p* values for the test are .19 for Denmark and .97 for Sweden, indicating that there is insufficient evidence to reject the null hypothesis that the data do not follow a monotonic trend. These results confirm the findings of the standard OLS regression model: the mean age at death of cohorts of Danish and Swedish centenarians has not been varying with the year of birth of the cohorts in our study. The evolution of maximum lifespans by birth year is shown in Fig. 2. In contrast to the mean, the maximum has been trending upward.

Even if the underlying distribution of deaths were invariant over time, the observed maxima would still tend to increase because of the growing population. This would complicate attempts to make inferences about individual lifespan increase. Can we analyze trends in the highest ages at death but still avoid use of the absolute maxima? What about trends in other parts of the distribution? Quantile regression is one way to do this because it is possible to explore the entire age at death distribution within a coherent framework.

### Quantile Regression

Although linear regression presents a parsimonious representation of the relationship between lifespan and cohort, it cannot provide important details. Quantile regression explores the entire range of the conditional age at death distribution. By considering the conditional age at death distribution at different points, we gain a more complete understanding of how age at death changes with cohort. It is also a more natural choice given that the interest is lifespan extension, which pertains to the right tail (or high quantiles) of the lifespan distribution.

**Fig. 3:**
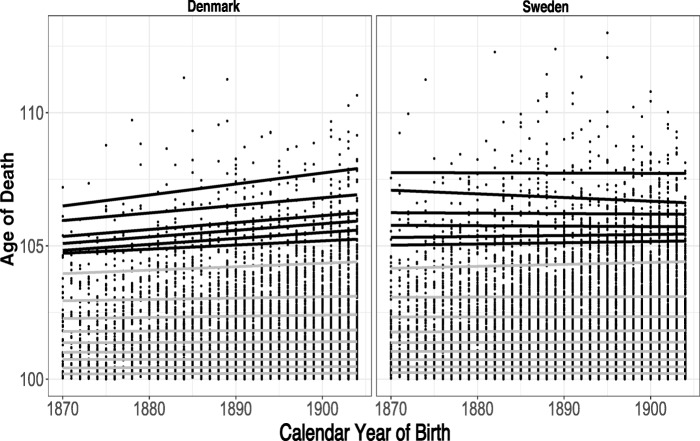
Data fitted with several quantile regressions. The 10th to 90th percentiles in increments of 10 % are shown in gray. The 94th, 95th, 96th, 97th, 98th, and 99th percentiles are shown in black. Each dot represents one individual.

Figure 3 presents the data fitted with several quantile regressions covering the entire distribution: the 10th to 90th percentiles (shown in gray) in increments of 10 %, plus the 94th, 95th, 96th, 97th, 98th, and 99th percentiles (the highest quantiles, shown in black). For our purposes, we consider the highest quantiles to be the tails of the distribution. The results for Denmark are in stark contrast to those for Sweden. For Sweden, there appears to be no meaningful relation between centenarian ages at death and cohort: most of the regression lines are close to horizontal, except the 98th percentile, which has a downward slope. Statistically, none of the fitted regressions are significant at the 5 % level. For Denmark, however, there is a trend: lifespan is related positively to cohort but only at the upper end of the distribution. The slopes of the lines over most of the distribution are flat but become apparent from the 90th percentile onward, and the strength of the relationship increases as lifespan becomes more extreme. For example, the age at death increases by about 0.022 years per one-unit increase in birth year at the 90th percentile; but by the 98th percentile, the rate of increase accelerates to 0.041 years per birth year.

It might be tempting to conclude that the upward trend in lifespan in Denmark could be due to a growing proportion of female centenarians over time. The proportion of female centenarians has been increasing over time in both countries, but this does not appear to have a serious impact on the results. When we control for sex (see the online appendix, section B), we observe similar patterns in the various quantiles. This dispels the notion that sensitivity to the sex ratio might be having a strong impact on the underlying trends and confirms the decision to combine males and females in the analysis to increase statistical power.

**Fig. 4:**
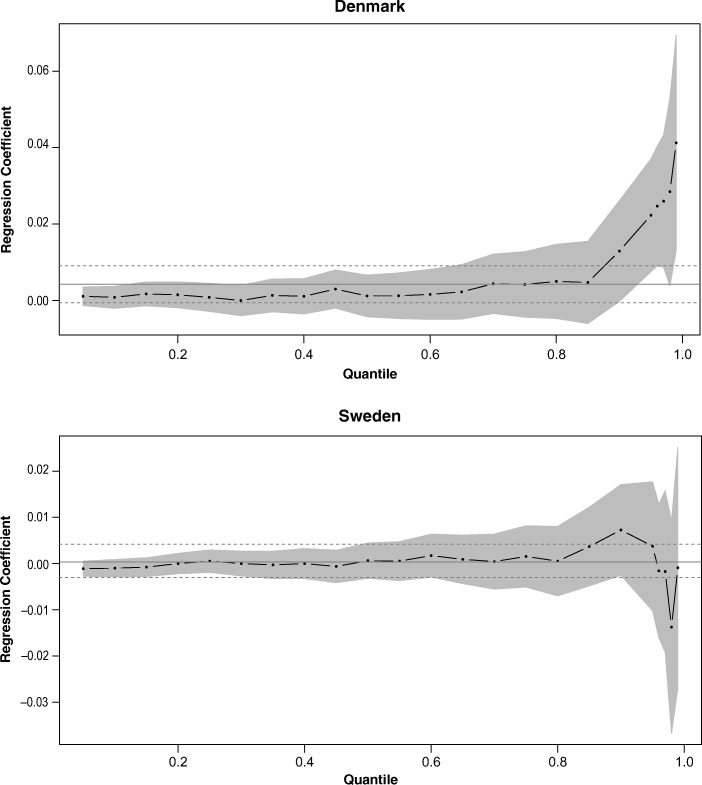
Estimated regression coefficients for different quantiles: from .05 to .95 in increments of .05 plus .96, .97, .98, and .99. The point-wise 95 % confidence band is shaded in gray. The solid horizontal line is the least squares regression coefficient of age at death on birth year, and the dashed lines are the 95 % pointwise confidence band about this trend.

An alternative graphical view of these regression results is shown in Fig. 4, illustrating the rate of change in lifespan with birth year over the entire centenarian lifespan distribution. The figure is particularly useful for visualizing how lifespan has been changing in the tails versus the rest of the distribution.

The confidence intervals (dashed lines) around the mean regression line envelops the horizontal line at 0, confirming the results of the linear regression: there is no trend in the mean. After the 85th percentile, the quantile regression coefficients show a continuous increase. However, only from the 94th percentile are they significantly different from 0 (at the 5 % level). Up to this point in the distribution, birth cohort is not a significant predictor of lifespan. Although the OLS regression coefficient may be a decent representation of how lifespan varies with birth year over most of the age range, it underestimates the strength of the relationship for the longest-lived centenarians in the Danish population. For the Swedish population, neither the mean nor the quantile regression coefficients are significantly different from 0, suggesting that centenarian lifespans have not been increasing in Sweden.

## Discussion

In this article, we present a methodology that enables the analysis of lifespan trends over time, defined in our data as the calendar year of birth. A key advantage to this method is that it avoids trends in the maximum ages at death and their associated theoretical issues. Inference for the maximum of a random variable is nontrivial, and more sophisticated approaches using extreme value theory (Fisher and Tippett 1928; Gumbel 1937; Medford 2017; Rootzén and Zholud 2017) are often necessary. As a proxy for the maxima, high quantiles are used. For human lifespan data, this should be a reasonable approach.

Studies of the highest lifespans can be challenging. Age is often misreported at older ages, and documentary evidence is sparse or absent altogether. Today’s centenarians were born more than 100 years ago, when record-keeping was less meticulous. The lack of reliable data at the highest ages has therefore restricted rigorous empirical studies to a handful of countries. The rising trend observed in Denmark is in line with previous empirical analyses from Japan, France, England and Wales, and the United States (Wilmoth and Lundström 1996; Wilmoth and Robine 2003; Wilmoth et al. 2000). Some recent studies have also suggested a more pessimistic view. Modig et al. (2017) indicated stagnation among Swedish centenarians, which is in line with the results of our quantile regression approach for Sweden. Using data from a larger collection of countries, Dong et al. (2016) and Vijg and Le Bourg (2017) controversially postulated that maximum lifespan might have peaked in the mid-1990s at about 115 years.

For Sweden, our analysis indicated no improvement in lifespan. Some possible reasons for this will be presented later, but henceforth, any methodological discussion will focus on the Danish results because those showed increasing lifespans, and we consider these of greater interest. For Denmark, the quantile regression approach is particularly useful because it unmasks the existing association at the ages in which we are interested: namely, the highest ages. Despite no lengthening of life among average—and indeed most—centenarians, improvement is seen among those who reach extreme ages. The highest lifespans have been trending upward over time, with the top 6 % (the 94th percentile) having a statistically significant trend. Furthermore, the rate of increase rises as the percentile increases. For example, the rate of increase in lifespan at the 99th percentile (0.041 years per year) is more than 2.5 times that at the 95th percentile (0.016 years per year).

**Fig. 5:**
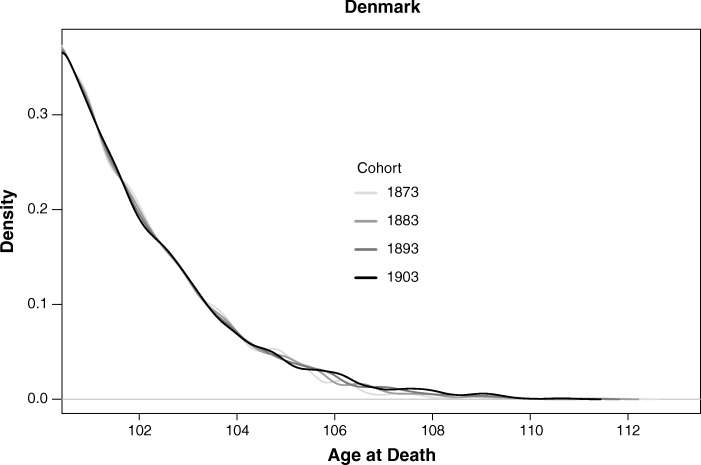
Conditional age at death density for birth cohorts 1873, 1883, 1893, and 1903.

Could these positive trends be driven by larger cohort sizes over time? To check this, we proceed as follows. First, we compute the conditional density function for the age at death from the values of the conditional quantile function, which we calculate in the quantile regression. We then repeat this procedure for several later birth cohorts. If the shapes of these densities are materially different from each other, it can be argued that the upward trend in the conditional quantiles (Fig. 3) is driven by the increasing size of successive cohorts. Otherwise, if the density estimates are reasonably similar, the increasing trend can be attributed to underlying longevity improvements. Figure 5 shows the smoothed density estimates for the 1873, 1883, 1893, and 1903 birth cohorts. Sampling variation apart, the shapes of the densities are very similar.

As an additional check of the influence of cohort size on the results, we rerun the quantile regression using larger cohorts comprising individuals randomly resampled from the existing data. We rerun the regression 5,000 times using bootstrapping and recalculate the coefficients. These results (online appendix, section C) demonstrate that using the larger sample sizes yields coefficients that are statistically indistinguishable. Because the increase in ages at death for the top quantiles is not attributable to larger cohorts, it must be attributed to improved survivorship.

It is often important in statistics to consider robustness to distributional assumptions. In this regard, quantile regression methods rank highly. They derive this robustness from the properties of basic sample quantiles. For example, given a data set that has some sample median, any data point above (below) the median can be changed to some other arbitrary value above (below) it without affecting the value of the median itself. So, given an estimate $\widehat{\upbeta}\left(\uptau \right)$ based on observations {*Age at Death*, *Birth Year*}, any ages at death could be altered, provided that the signs of the residuals are unchanged. To illustrate this property, we apply a ceiling of 108 years (the lowest allowable for this data) to the lifespans and recalculate the regression coefficients. As expected, we see no change (figure not shown). This result adds further credibility to the observed quantile trends.

For the 1870–1904 birth cohorts, we find an increase in age at death among the longest-lived Danish centenarians but no increase for those in Sweden. Although these two Scandinavian countries are similar in many ways, Denmark has generally experienced higher mortality (lower lifespans) than Sweden, and this excess mortality has been attributed to smoking (e.g., Lindahl-Jacobsen et al. 2016; Sundhedsministeriet (Danish Health Ministry) 1994). During the early to mid-1990s, smoking in Denmark as well as alcohol consumption and sedentary lifestyle decreased, leading to higher life expectancies. However, the effect of these factors on centenarians is unclear. Around the same time, Denmark also attempted to close the mortality gap with Sweden. Increased financial support and initiatives targeted better treatment of cardiovascular and other diseases (Christensen et al. 2010). Even though these programs did not specifically target the oldest-old, we speculate that some benefits spilled over to them.

The diverging trends in lifespan between the two countries could be due to different levels of health among their respective elderly populations. Recent studies have shown improvements in health as measured by activities of daily living (ADL) in cohorts of female centenarians in Denmark (Engberg et al. 2008; Rasmussen et al. 2018) among the 1895, 1905, and 1915 cohorts. Because women heavily outnumber men at the advanced ages, the overall contribution is positive. In Sweden, trends in health for the elderly have been less optimistic. A study using multiple health indicators of Swedes aged 77 and older between 1992 and 2002 found indications of worsening health from the mid-1990s onward—both a lack of improvement in ADL and deterioration in mobility, cognition, and performance tests (Parker et al. 2005, 2008).

In previous studies of oldest-old mortality among several developed countries, Kannisto (1994, 1996) argued that the observed declines in mortality at the highest ages (as opposed to, say, middle ages) were driven more by period than cohort effects. These period effects have an immediate impact on mortality patterns at the time they occur. While Denmark committed more time and resources in attempts to improve life expectancy, Sweden experienced a serious economic crisis in the early 1990s, which led to fiscal consolidation and reduced spending on public services, including health care for the elderly. At the same time, a deinstitutionalization of inpatient elder care was already underway, with a shift away from hospitals to nursing homes and a reduction in the number of nursing home beds. The economic crisis might not have directly caused this shift, but it certainly accelerated its implementation. The cost cuts have left some older people at risk, particularly those in the lower socioeconomic groups. Since that time, Denmark and Sweden have had disparate approaches to elder care. Sweden has adopted an approach targeting the most frail, whereas Denmark continued with generous provision of care, with the obvious result that services reach a higher proportion of the elderly population. Some studies have suggested that Sweden’s approach may have resulted in the lack of care for some who require it and that the least well-off segments of the elderly population rely more heavily on family care, which may be of lower quality (e.g., Rostgaard and Szebehely 2012).

In summary, only those who have already attained the highest ages appear to have lifespans that are improving over time—and only in Denmark. Persons who attain age 100 are a select group, but there appears to be an additional layer of selection favoring a super-select group. Those within this super-select group are obviously the most durable, and it is they who are experiencing longer lives. Perhaps because of their inherent resilience and particular physiology, they are best able to benefit from the advances in health care. Unfair maybe, but the vagaries of human survival appear to favor winners: only the very oldest are growing older.

## Electronic supplementary material

ESM 1 (PDF 1119 kb)
