# Supplementary material for: A Cohort Comparison of Lifespan After Age 100 in Denmark and Sweden: Are Only the Oldest Getting Older?
Source: Demography. 2019 Jan 18;56(2):665–77. doi: 10.1007/s13524-018-0755-7 (PMC6449298; doi:10.1007/s13524-018-0755-7)
Supplement: Supplementary file 1 — (PDF 1119 kb) [file 13524_2018_755_MOESM1_ESM.pdf]

Online Appendix

A. Quantile regression basics

Suppose Y is the response variable, and X is the p-dimensional predictor. Let $F_Y(y|\mathbf{X} = x) = P(Y \leq y|\mathbf{X} = x)$ be the conditional cumulative distribution function (CDF) of Y given $\mathbf{X} = x$. Then the τth conditional quantile of Y is defined as the inverse of the CDF or mathematically,

$$Q_\tau(Y|\mathbf{X} = x) = \inf\{y : F(y) \geq \tau\}$$

This can be extended to the General Linear quantile regression model:

$$Q_\tau(Y|\mathbf{X} = x) = \mathbf{X}^T\boldsymbol{\beta}(\tau), \quad 0 < \tau < 1,$$

where $\boldsymbol{\beta}(\tau) = (\beta_1(\tau), \ldots, \beta_p(\tau))^T$ is the quantile coefficient that may depend on τ and represents the marginal change in the τth quantile due to the marginal change in x.

Whereas the linear regression coefficients are solved by minimising least squares,

$$\hat{\beta} = \arg\min_{\beta \in \mathbf{R}^p} \sum_{i=1}^{n} (y_i - \mathbf{x}_i^T \beta)^2$$

the τth regression quantile of the linear conditional quantile function $Q_\tau(Y|\mathbf{X} = x)$ is estimated by minimizing a weighted sum of the absolute deviations,

$$\hat{\beta}(\tau) = \arg\min_{\beta \in \mathbf{R}^p} \sum_{i=1}^{n} \rho_\tau(y_i - \mathbf{x}_i^T \beta)$$

for any quantile $\tau \in (0, 1)$. For example the case $\tau = 0.5$ corresponds to median regression.

B. Quantile regression by male/ female

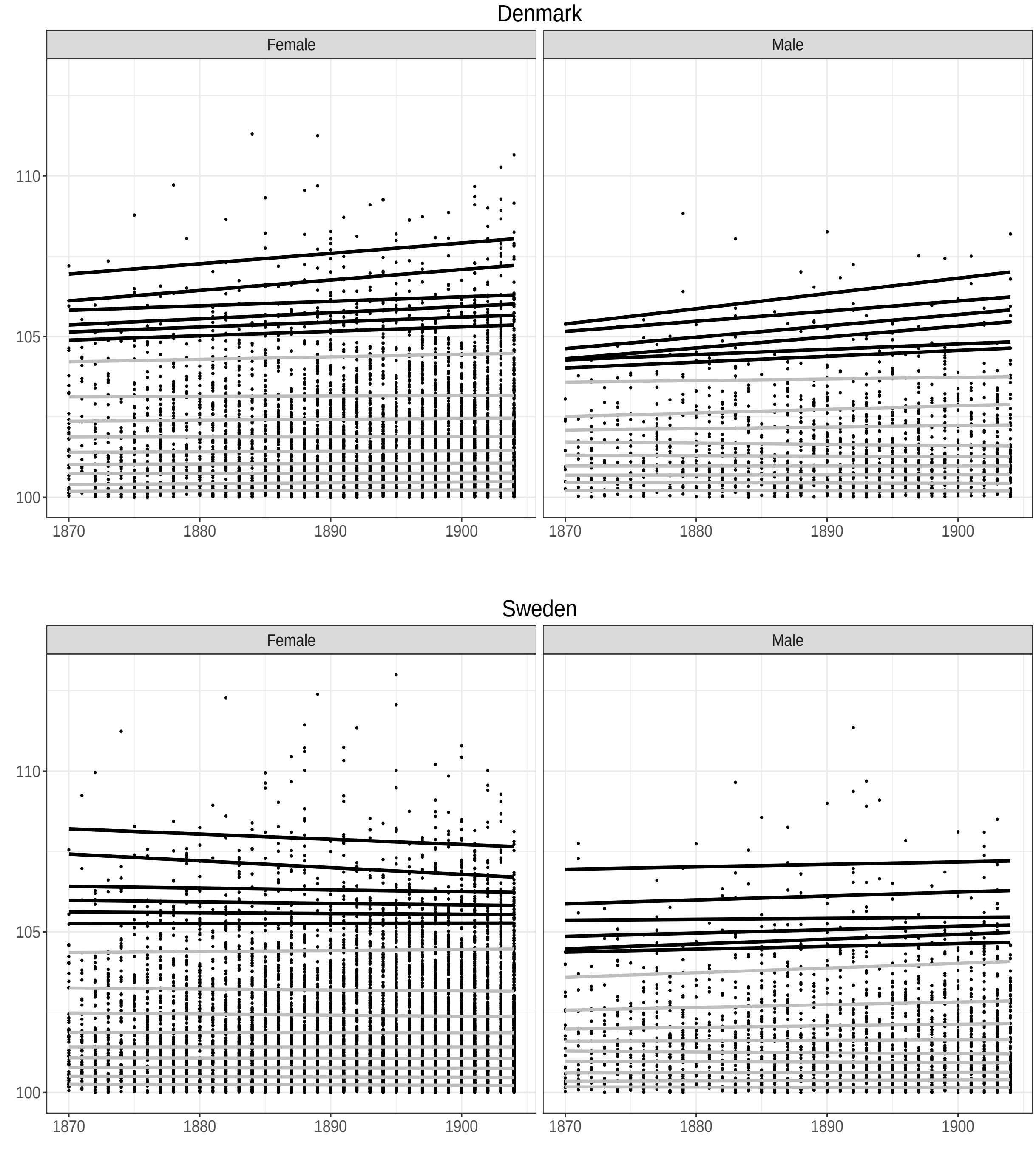

Figure A1: Data fitted with several quantile regressions for females and males, separately. In grey: the 10th to 90th percentiles in increments of 10%. In black: the 94th, 95th, 96th, 97th, 98th and 99th percentiles. Each dot represents one individual.

C. Growth of cohort sizes and check of increase sample size on estimated regression coefficients

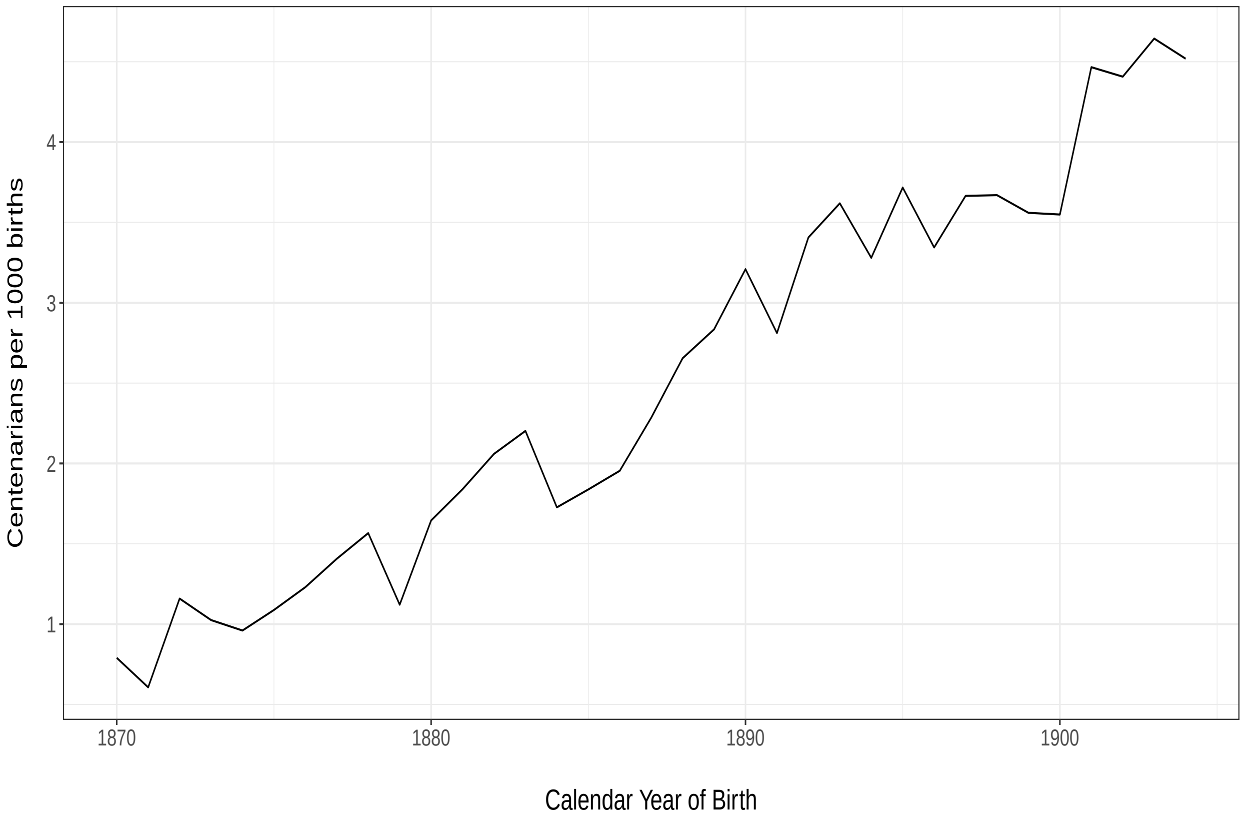

Figure A2: Proportion of each birth cohort (1870-1904) in Denmark attaining age 100.

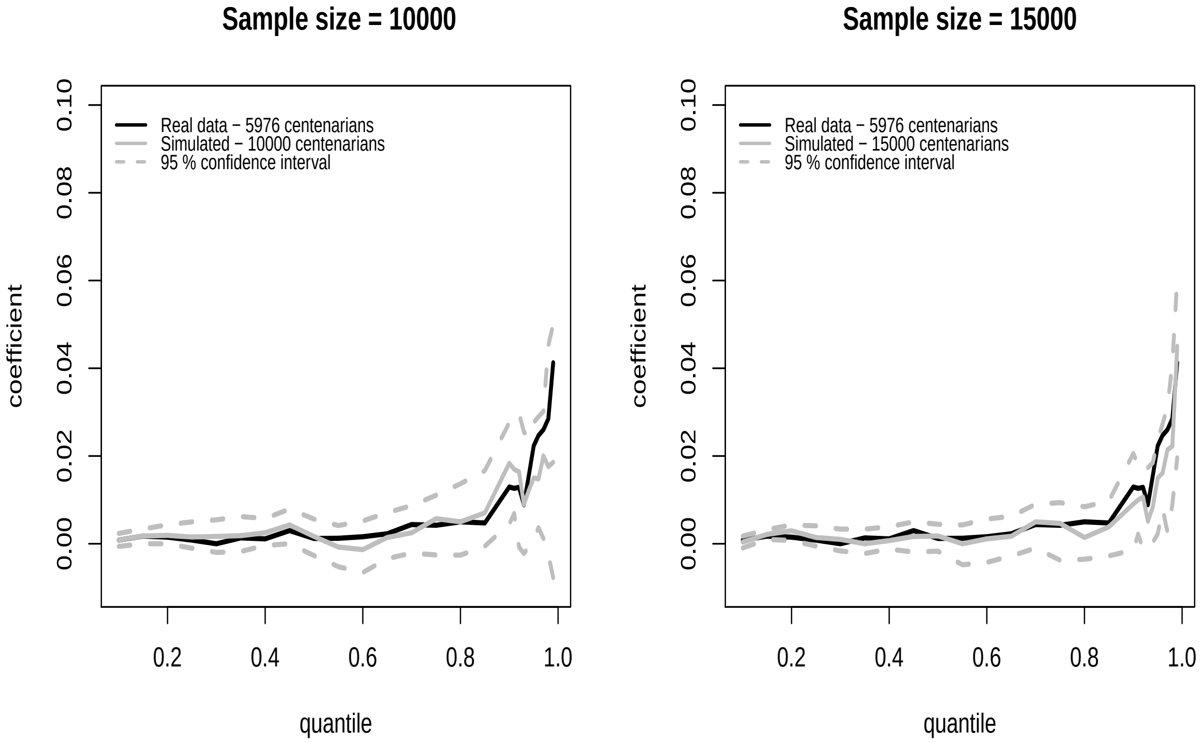

Figure A3: Hypothetical sample sizes of size 10000 and 15000 with bootstrapped regression coefficients and 95% confidence intervals.
